# Mortality risks of body mass index and energy intake trajectories in institutionalized elderly people: a retrospective cohort study

**DOI:** 10.1186/s12877-022-02778-1

**Published:** 2022-01-31

**Authors:** Yoshiaki Kawakami, Jun Hamano

**Affiliations:** 1 Department of Nursing, Tokyo Ariake University of Medical and Health Sciences, Tokyo, Japan; 2 Division of Clinical Medicine, Faculty of Medicine, University of Tsukuba, Tsukuba, Japan

**Keywords:** Body mass index, Mortality, Elderly, Energy intake, Long-term care, Unintentional weight loss

## Abstract

**Background:**

Many factors can identify the mortality risks of institutionalized elderly people; among all such factors, body mass index (BMI) and energy intake (EI) can be employed as independent predictors. The objective of this study was to elucidate about the mortality risks and the trajectory of two parameters obtained from continuously monitored data.

**Methods:**

This retrospective cohort study targeted 218 elderly people who were admitted to a nursing home for at least 6 months between 2007 and 2020 and passed away at the nursing home. BMI and EI (kcal) per body weight (BW) were continuously measured until death.

**Results:**

BMI continued to decrease for 5 years until death. The rate of change of BMI significantly increased for 3 years before death (*P* = 0.004). In contrast, the rate of change of EI per BW significantly increased after 1 year before death (*P* < 0.001); in addition, 2 months before death, this rate of change significantly exceeded that of BMI (*P* = 0.007). In the four risk groups that were examined, a BMI of <18.0 + EI per BW of <29.2 and a BMI of <17.3 + EI per BW of <28.3 were significantly correlated with a high risk of death (log-rank test: *P* < 0.001, *P* = 0.002, respectively). There was no significant difference in the hazard ratio based on the age at the time of admission; however, when EI per BW was <23.8, the risk of death was significantly higher [hazard ratio = 4.36; 95% confidence interval: 2.31–8.24].

**Conclusions:**

Elderly people in the current study presented a tendency toward decreasing BMI starting 60 months prior to death even if EI per BW remained constant. In the 1 year before death, EI per BW rapidly decreased. When the rate of change of EI per BW exceeded the rate of decrease of BMI, it was considered to be the point of no return when death was imminent. Our study showed that identifying mortality risks from the relationship between the trajectories of the two parameters that were continuously measured for several months to years is possible.

## Background

Although many families of elderly people and experts in the field of elderly care believe that palliation is an important goal for institutionalized elderly people, elderly people who pass away in a facility do not receive ideal palliative care [1]. It is possible that because advanced dementia and debility, when not specified otherwise, are not considered terminal conditions, care might not be directed toward optimum comfort until death is acknowledged as being imminent [2, 3]. Family members providing care are not receptive of the idea that their relatives are in the dying phase, whereas healthcare professionals do not communicate definitive evidence indicative of the approaching death. This is a hindrance to palliative care [4–7].

Therefore, it is extremely important to make an accurate risk assessment of the prognosis and imminent death. Mortality prediction and risk assessment of elderly people requires the analysis of many factors, such as blood parameters, the number of comorbidities, immune dysfunctions, and conducting statistical approaches [8–10]. However, such data collection and evaluation require skilled personnel, a multidisciplinary approach, and in some cases, high costs. Thus, the necessary data may not be available for institutionalized elderly people such as those admitted to a nursing home (NH) or in a homecare setting [7]. Factors for mortality prediction and risk assessment include specific causes and the number of comorbidities; however, institutionalized elderly people usually have multiple morbidities [11] but do not receive a routine workup, making it difficult to identify the true cause of their death [12].

Nutritional status is significantly or independently associated with mortality [13–15]. Therefore, the mortality risks associated with nutritional status could be used to predict mortality on their own. However, institutionalized elderly people with dementia experience mental and cognitive impairments, psychological conditions, and dysphagia, presenting complex nutritional problems in the early stage of death [16, 17]; thus, even if mortality was predicted, predicting the timing of death would be difficult.

Many studies have shown that a decrease in body weight (BW) and body mass index (BMI) increases the risk of mortality [18–22]. It has particularly been verified that unintentional weight loss in elderly people increases the risk of death [23]. According to our 5-year longitudinal study of 106 elderly people who were admitted to and died in an NH, even if the energy intake (EI) remained constant, BMI started to gradually decrease 5 years prior to death, and EI significantly decreased several months prior to death [24]. In other words, when the trajectory of EI rapidly decreases compared with the trajectory of decreasing BMI, it is reasonable to consider it as the “point of no return,” where death is imminent. An evaluation of the relationship between the two trajectories might be a useful tool in identifying mortality risks.

Thus, this study aimed to understand the mortality risks by elucidating about the relationship between the trajectory of BMI and the trajectory of EI, which are parameters that could be routinely monitored for institutionalized elderly people, to determine whether they could be used to make an accurate mortality prediction and risk assessment for elderly people.

## Methods

### Study Design and Participants

The present study was a single-center retrospective cohort study. Baseline data collection was conducted from April 1, 2007 to February 29, 2020. The subjects were all elderly individuals (aged ≥65 years) who died during this period in “Junseien,” which is an NH for the Elderly, located in Kanagawa Prefecture in Japan. They were officially identified as being in a state requiring long-term care according to the Public Nursing Care Insurance Law. Residents of a facility covered by the Public Nursing Care Insurance, such as this NH, are required to be officially recognized as being at the care-need level 3 or above as per the Long-Term Care Insurance system.

The clinical picture of care-need level 3 individuals is as follows: difficulty in rising, moving, and transferring to and from a vehicle on their own, understanding the routine schedule [25], and remembering what they were doing prior to an interview. At the highest level, care-need level 5, in addition to the abovementioned conditions, includes dysphagia, disorientation, limited range of motion in the joints, motor paralysis, and other problems [26, 27].

All subjects received professional care assistance under the same living conditions from the time of admission. They consumed three main meals (breakfast, lunch, and dinner) that were managed as per their EI (kcal) and nutrient requirements by registered dieticians of the facility at a given time. All their meals were cooked and arranged at the facility. If they had difficulty in eating or swallowing, they received assistance.

The subjects included elderly individuals who continuously resided in the NH for at least 6 months and who passed away during their stay without receiving artificial nutrition and hydration (ANH). The exclusion criterion was elderly individuals who died after being hospitalized due to acute diseases or symptoms requiring continuous medical care that the NH could not provide.

Among the 267 individuals who passed away at the NH during the survey period, 218 met the inclusion criteria and were included in the present study.

### Clinical Measurements

Nurses and trained caregivers measured the height of all elderly individuals to 0.1 cm and BW to 0.1 kg following a standard protocol [28] within 72 hours after admission and every month thereafter. For those with difficulty in standing up straight, the height was measured to 0.1 cm using a measuring tape and the weight was measured using a wheelchair scale.

A registered dietitian calculated the calorie count for each meal for each subject and placed the meal on an individual tray. Upon completion of each meal, the trained caregivers observed and recorded the proportion of meals consumed on a scale of 1–10. EI (kcal) was calculated daily by multiplying the proportion of the meal consumed by the nutritional value of each meal.

### Statistical Analysis

The participants of this study were divided into four groups: 6 months to <12 months, 12 months to <36 months, 36 months to <60 months, and >60 months based on the number of months they survived following their admission. Because the age at admission and death, sex, height/weight at admission, BMI, mean EI per day 1 month after admission, and daily EI per BW, which also had an impact on survival [29, 30], were used to evaluate the energy consumption of the elderly people who stayed in a long-term care setting for a long period of time, we calculated the mean EI per BW and checked for statistically significant differences using one-way analysis of variance.

Furthermore, up to 60 months immediately before death, we calculated the mean monthly BMI and the mean daily EI per BW for every month. Later, we calculated the rate of change compared with the mean value 6 months ago. For each rate of change, we checked for statistically significant differences in the rate of change over 12 months (every 12 months from 60 months prior to death) using the paired t-test. This was followed by a verification of the change in the mean rate of change between the two parameters for each month during the 12 months prior to death.

Finally, based on BMI and EI per BW data from 12 months and 6 months prior to death, we divided the subjects into four groups at the time of admission, plotted Kaplan–Meier curves, and performed associated log-rank tests. Risk factors for mortality were assessed using the Cox proportional hazards regression model and associations were presented as hazard ratios (HRs) and 95% confidence intervals (CIs).

A *P*-value of <0.05 was considered statistically significant. All statistical analyses were performed using JMP Pro 15.2.0 (for Windows 10) (SAS Institute Inc., Cary, NC, USA).

## Results

The baseline characteristics of the 218 elderly people are presented in Table 1. Subjects were divided into four groups: survival of 6 months to <12 months, 12 months to <36 months, 36 months to <60 months, and >60 months. There was a significant difference in the age at death, sex, height, BMI, EI, and EI per BW (Table 1).

**Table 1:** Baseline clinical characteristics of the 218 elderly people by the survival duration (months)

	6 to <12 months			12 to <36 months			36 to <60 months			≥60 months			*P*-value *
	*n* = 29			*n* = 85			*n* = 53			*n* = 51			
Age at admission (years)	87	±	6	86	±	8	86	±	8	85	±	7	0.9091
Age at death (years)	87	±	7	88	±	8	90	±	8	91	±	7	0.0205
Women (%)	59			78			83			90			0.0105
Height (cm)	156	±	9	152	±	8	150	±	8	150	±	7	0.0022
BW (kg)	44.6	±	7.5	42.7	±	8.3	42.4	±	8.2	45.9	±	9.0	0.1003
BMI (kg/m^2^)	18.3	±	2.7	18.5	±	3.0	18.8	±	2.7	20.5	±	3.9	0.0022
EI (kcal/day)	1,055	±	345	1,256	±	235	1,241	±	271	1,385	±	191	<0.0001
EI per BW (kcal/kg/day)	24.0	±	8.2	30.3	±	7.6	29.8	±	6.8	31.2	±	6.8	0.0003

Data are the mean ± standard deviation or n (%)

^*^P-value for one-way analysis of variance. Sex was analyzed using the Fisher’s exact test

*BMI* Body mass index, *BW* Body weight, *EI* energy intake

**Fig. 1:**
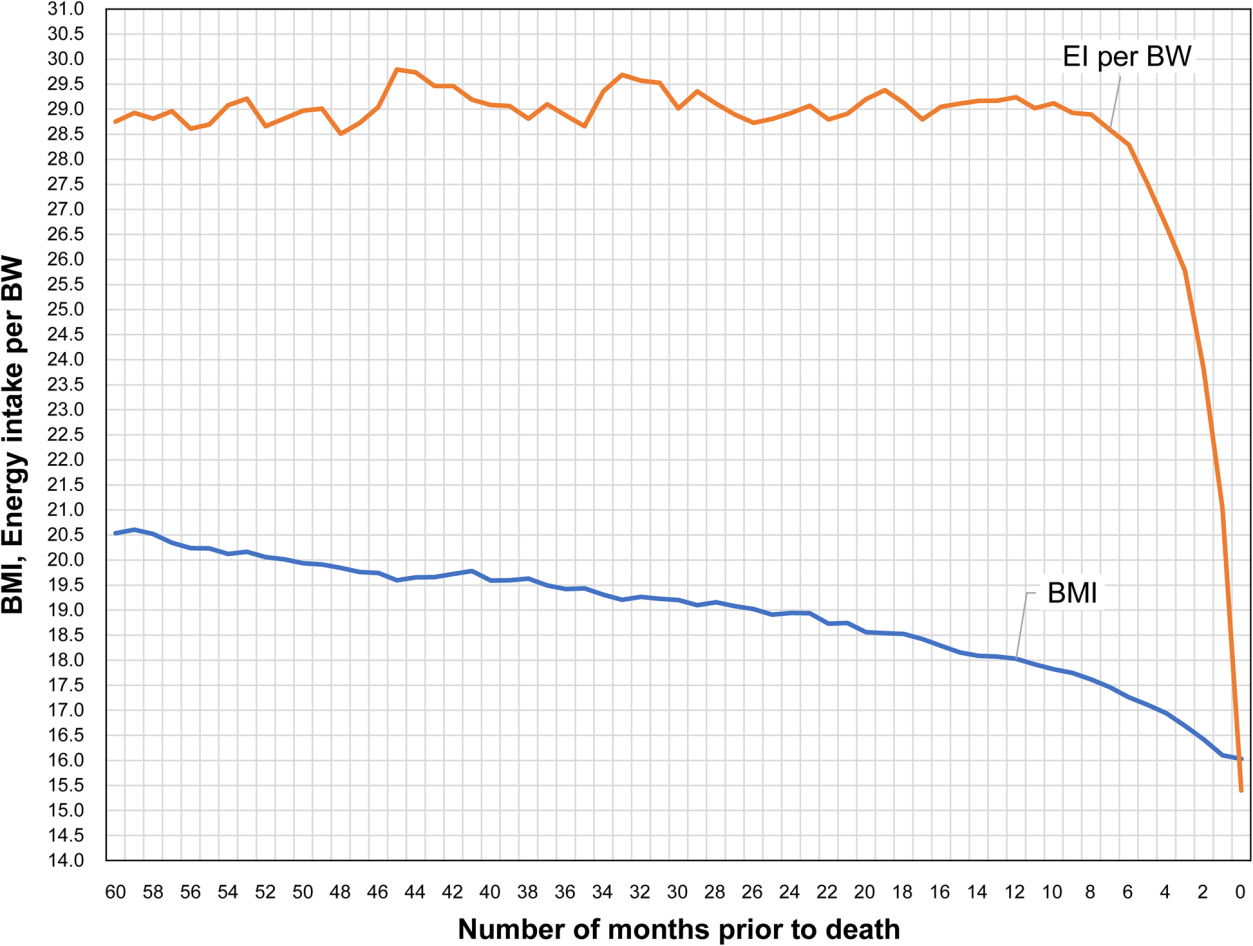
Changes in the mean BMI and EI per BW 60 months prior to death

The trends observed for the two parameters and the relationship between them were determined (Fig. 1).

Table 2 shows the difference in the rate of change of BMI and EI per BW for 60–48 months prior to death, 48–36 months prior to death, 36–24 months prior to death, 24–12 months prior to death, and 12 months to immediately prior to death. BMI continuously decreased for 60 months prior to death, but its decrease was significant from 36 months prior to death. There was no significant change in EI per BW until 12 months prior to death, following which there was a significant decrease in this parameter immediately before death.

**Table 2:** Difference in the rate of change in BMI and EI per BW every 12 months from 60 months prior to death

	BMI			EI per BW		
Months prior to death	Mean difference	SD	*P*-value*	Mean difference	SD	*P*-value*
60–48	−0.26	0.25	0.2907	−0.76	0.68	0.2666
48–36	−0.24	0.17	0.1692	0.27	0.68	0.6915
36–24	−0.44	0.15	0.0042	−0.18	0.63	0.7822
24–12	−0.94	0.15	<0.0001	0.00	0.49	0.9931
12–prior to death	−2.20	0.26	<0.0001	−13.21	1.22	<0.0001

^*^P-value for paired-sample t-test

*BMI* Body mass index, *BW* Body weight, *EI* Energy intake

We obtained the difference between the rates of change of the two parameters, BMI and EI per BW, for each month from 12 months prior to death in Table 3. After EI per BW began to decrease 7 months prior to death, and particularly in the 4 months prior to death, when the decrease in EI per BW exceeded that of BMI, we verified this change using the paired t-test. The mean rate of change of EI per BW that exceeded that of BMI 4 months prior to death continued to decrease and was significantly higher than that of BMI 2 months prior to death.

**Table 3:** Rate of change of BMI and EI per BW for each month from 12 months prior to death

		BMI		EI per BW			
Months prior to death	Mean	Mean rate of change	SD	Mean	Mean rate of change	SD	*P*-value*
12	18.0	0.970	0.069	29.2	1.020	0.224	0.0117
11	17.9	0.971	0.062	29.0	1.038	0.305	0.0111
10	17.8	0.974	0.060	29.1	1.015	0.187	0.0147
9	17.7	0.972	0.060	28.9	1.011	0.244	0.0477
8	17.6	0.971	0.063	28.9	1.015	0.323	0.0676
7	17.5	0.965	0.067	28.6	0.988	0.202	0.1569
6	17.3	0.959	0.065	28.3	0.978	0.193	0.2033
5	17.1	0.953	0.072	27.5	0.973	0.268	0.3364
4	16.9	0.951	0.069	26.7	0.944	0.242	0.6511
3	16.7	0.943	0.077	25.8	0.932	0.303	0.5732
2	16.4	0.935	0.077	23.8	0.865	0.359	0.0074
1	16.1	0.926	0.086	21.0	0.774	0.368	<0.0001
Prior to death	16.0	0.921	0.093	15.4	0.594	0.395	<0.0001

^*^P-value for paired-sample t-test

*BMI* Body mass index, *BW* Body weight, *EI* Energy intake, *SD* Standard deviation

**Fig. 2:**
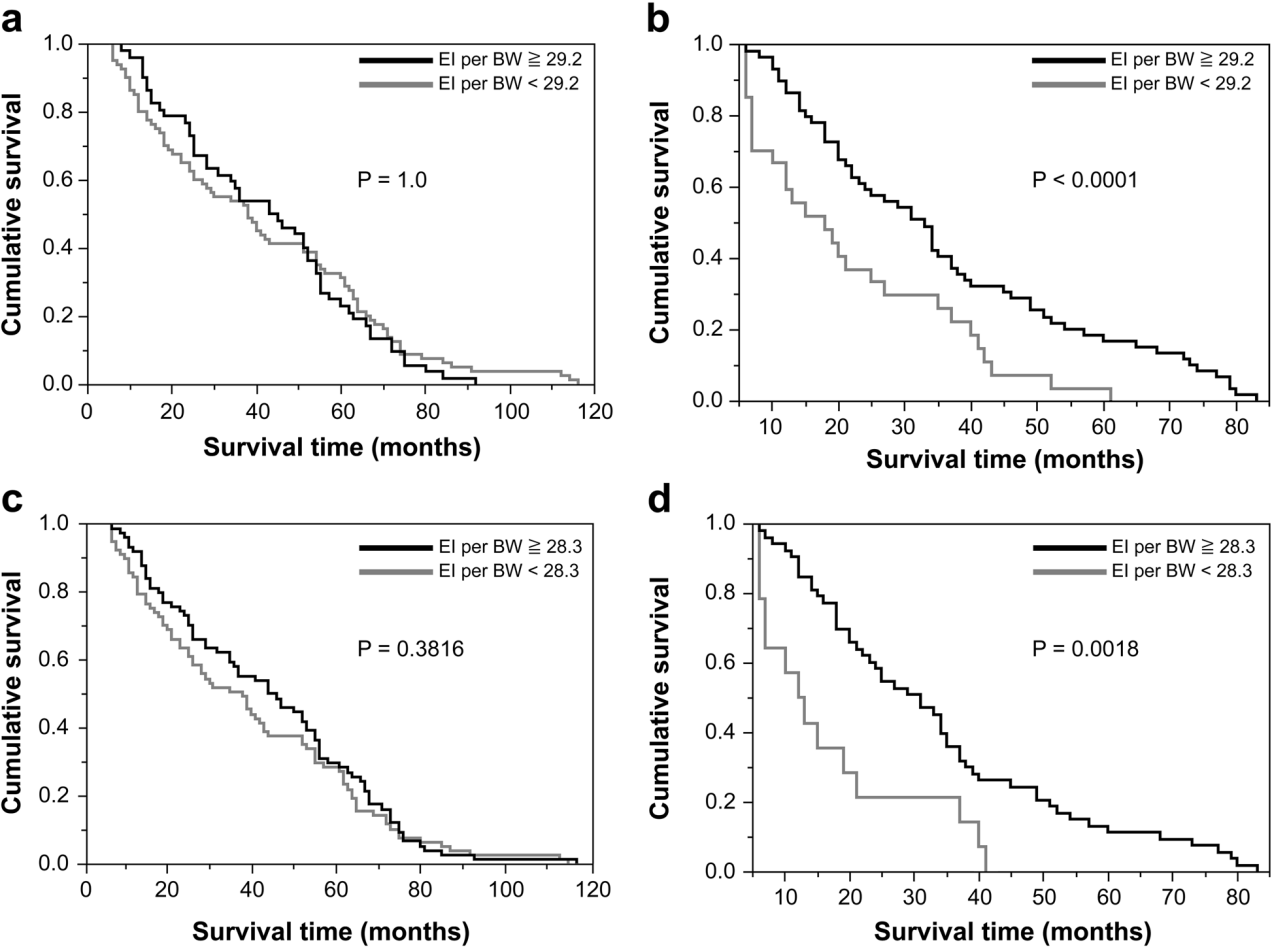
Survival curves from the time of admission. **a** BMI ≥18.0 Groups with EI per BW ≥29.2 (*n* = 80) and of <29.2 (*n* = 52) **b** BMI <18.0 Groups with EI per BW ≥29.2 (*n* = 59) and of <29.2 (*n* = 27) **c** BMI ≥17.3 Groups with EI per BW ≥28.3 (*n* = 74) and of <28.3 (*n* = 77) **d** BMI <17.3 Groups with EI per BW ≥28.3 (*n* = 53) and <28.3 (*n* = 14)

BMI and EI per BW were 18.0 and 29.2, respectively, at 12 months prior to death and 17.3 and 28.3, respectively, at 6 months prior to death. Based on these data, we divided subjects into four groups, namely, those whose scores were the same or above the mean and those whose scores were below the mean at the time of admission; plotted the Kaplan–Meier curves; and performed associated log-rank tests. In the group with BMIs of <18.0 and EIs per BW of <29.2 and the group with BMIs of <17.3 and EIs per BW of <28.3, the survival rate decreased significantly (Fig. 2).

Finally, based on the sex and age at the time of admission and values obtained from each mean of BMI and EI per BW at 12, 6, and 2 months prior to death, we created four categories of BMI—<16.4, 16.4 to <17.2, 17.3 to <17.9, and ≥18—and four categories of EI per BW—<23.8, 23.8 to <28.2, 28.3 to <29.2, and ≥29.2—and obtained the HRs and their 95% CIs using the Cox proportional hazards model (Table 4).

**Table 4:** Characteristics of subjects and hazard ratios and 95% confidence intervals for each factor

Characteristics		Hazard ratio	95% confidence interval		*P*-value
Sex					
	Male	1.77	1.24	2.53	0.0016
Age (years)					
	<74	1.00	0.51	1.95	0.9967
	75–84	0.69	0.42	1.14	0.1482
	85–94	0.87	0.54	1.41	0.5687
	>95	1	Reference		
sBMI					
	<16.4	1.73	1.17	2.55	0.0057
	16.4–17.2	2.02	1.25	3.26	0.0042
	17.3–17.9	1.14	0.69	1.90	0.6028
	>18	1	Reference		
EI per BW					
	<23.8	4.36	2.31	8.24	<0.0001
	23.8–28.2	1.18	0.85	1.65	0.3180
	28.3–29.2	1.11	0.63	1.95	0.7229
	>29.2	1	Reference		

*BMI* Body mass index, *BW*, Body weight, *EI* Energy intake

The results showed that the male subjects had an HR of 1.77 (95% CI: 1.24–2.53), indicating that the risk of death was 1.77 times significantly higher than that for the female subjects. The group with a BMI of <16.4 had an HR of 1.73 (95% CI: 1.17–2.55), indicating that compared with the group with a BMI of ≥18.0, the risk of death was 1.73 times significantly higher. The group with a BMI of 16.4 to <17.2 had an HR of 2.02 (95% CI: 1.25–3.26), indicating that the risk of death was 2.02 times significantly higher than that of the group with a BMI of ≥18.0.

When the EI per BW was <23.8 kcal/kg, the HR was 4.36 (95% CI: 2.31–8.24), indicating that the risk of death was 4.36 times significantly higher than that when EI per BW was ≥29.2 kcal/kg. Comparisons based on the age at the time of admission did not present significant HRs.

## Discussion

To our knowledge, this is the first study to have deciphered the trajectories that longitudinally examined BMI for each month until death and calculated EI per BW from three daily meals during these months (up to 5 years prior to death) for elderly people who died at an NH.

The most notable finding in this study was that the changes of BMI and EI per BW showed distinctive trajectories over time as subjects came closer to their death.

BMI of elderly people who died at the NH gradually decreased from 60 months prior to death and continued to decrease until death. The mean BMI at 60 months prior to death was 20.5, but the mean BMI immediately prior to death was 16.0. In a cross-sectional survey, a BMI of 20.0 was considered a valid threshold for determining a high risk for short-term mortality in elderly individuals [21, 31]. Furthermore, the World Health Organization classifies adults aged ≥20 with BMIs <18.5 as being underweight. The mean BMI of the elderly subjects in this study was <20.0 at 50 months prior to death and was <18.5 at 17 months prior to death, clearly continuing to follow a decreasing trajectory. Therefore, by analyzing not only the specific value of BMI but also its trajectory for several months to years, the long-term mortality risk over a longer span (i.e., years) could be determined.

EI per BW of elderly people did not show any significant change in trend until 12 months prior to death. EI per BW began to decrease at 7 months prior to death, then followed an irreversible decreasing trend. Low EI per BW is implicated in mortality and is an independent predictor of all-cause mortality [32]. If BMI is useful in identifying the long-term mortality risk, a rapid decrease in the trajectory of EI per BW and a change in its value to <23.8 represents a short-term mortality risk within several months, which is a useful indicator.

The second important finding was that the trajectories of the changes in BMI and EI per BW do not decrease in parallel at similar rates of change.

At 60 months prior to death, BMI showed a decreasing trajectory, but EI per BW either continued to increase until 12 months prior to death or did not show continuous decrease, not presenting a notable difference in the rate of change. EI per BW for the subjects in the present study at 12 months prior to death was 29.2 kcal/BW, which was higher than 23.6–23.8 kcal/BW calculated based on the Harris–Benedict equation for calories that are necessary for each day [33]. In other words, despite the intake of the required amount of calories, BW continued to decrease. The rate of change of EI per BW was greater than BMI at 4 months prior to death and significantly more than the rate of change of BMI at 2 months prior to death. Therefore, following a long-term BMI loss and stable EI per BW, a relationship wherein the depletion of EI per BW increases faster than the declined in BMI indicates an imminent death.

In past studies, BMI was examined [31, 34, 35] as a factor related to mortality [36, 37] and malnutrition, and mortality was discussed based on BMI measured at a point in time. However, our study showed that by continuously measuring BMI and EI and examining the resulting trajectories and their relationship, instead of treating each parameter as a separate predictor, mortality risks could be identified with more accuracy. Risk assessment based on the relationship between the trajectories of BMI and EI per BW is an effective way of detecting mortality among institutionalized elderly individuals.

An interesting aspect of the present study is that by examining the relationship between the trajectories of the two parameters, it is possible to evaluate the time-imminent risk of death. When the rate of change of BMI significantly decreases while that of EI per BW does not, mortality risks tend to increase within 3 years. Under long-term BMI loss, when the nutritional depletion becomes evident and the rate of change of EI per BW significantly exceeds that of BMI, it is the point of no return and death will occur within 1–2 months. Assuming such a trajectory, we reckon that when BMI and EI per BW reach critical levels, such as 18.0 and 29.2 or 17.3 and 28.3, respectively, the chance of survival diminishes dramatically.

It is considered necessary to maintain a constant EI as a measure to prevent the decrease in weight and BMI of elderly people who are admitted to an NH [38, 39]. Specifically, in the case of an elderly person with dementia who has not expressed an intention to end life care, a question of whether to implement ANH or not puts pressure on families and the NH staff [40–42]. According to the results of the present study, when BMI decreases regardless of sufficient EI per BW, despite the deficiency in EI being supplemented by additional energy intake, such as ANH, the maintenance of body weight or BMI is predicted to be difficult. We consider palliation to be the most important goal for institutionalized elderly people, and a decrease in EI following a decrease in BMI must be accepted as a transitional phenomenon to death.

The present study has certain limitations. First, the sample size was slightly small and limited to Japanese people from a single NH; therefore, some results may only be applicable to the Japanese population. We also excluded elderly people who died within 6 months of being admitted to the NH; thus, the results of this study cannot be applied to elderly people with acute diseases requiring continuous medical care or acute symptoms for which the NH could not provide adequate care.

Furthermore, the present study targeted all elderly people who lived and died in the NH under the same living conditions for approximately 13 years without ANH. These elderly people mostly spent their time in a chair or on a bed and lacked the mobility or will to go to restrooms on their own. For 1 year prior to death, their BMIs dropped by a mean value of 2.0 accompanied by unintentional weight loss of approximately 5 kg; thus, all the subjects in the present study were considered to be frail elderly individuals [43]. Therefore, mortality risks for frail elderly individuals with advanced dementia and debility not otherwise specified could be identified using the present results.

## Conclusion

Dying institutionalized elderly people may have constant EI per BW values but decreasing BMIs from approximately 60 months prior to their death. Once such individuals reach a period of several months before death, EI per BW rapidly decreases. When the rate of decrease of EI per BW exceeds that of BMI, it is considered the point of no return and that death is imminent. Therefore, longitudinal observations of trajectories of BMI and EI over several months to years and the analysis of the relationship between these trajectories could identify mortality risks.

## Data Availability

The data cannot be shared publicly because there was no such approval in the study protocol. The datasets used and analyzed during the study are available from the corresponding author upon request and subject to ethical approval.

## Abbreviations

BW: Body weight
BMI: Body mass index
EI: Energy intake
NH: Nursing home
ANH: Artificial nutrition and hydration
